# Investigating the effect of wearing the hijab: Perception of facial attractiveness by Emirati Muslim women living in their native Muslim country

**DOI:** 10.1371/journal.pone.0199537

**Published:** 2018-10-05

**Authors:** Mercedes Sheen, Hajar Aman Key Yekani, Timothy R. Jordan

**Affiliations:** 1 Department of Psychology, Zayed University, Dubai, UAE; 2 Department of Psychology, Middle East Technical University, Northern Cyprus Campus, Northern Cyprus; Saint Peter's University, UNITED STATES

## Abstract

The Hijab and other forms of Islamic veiling are important social, cultural, and religious symbols that are central to the identity of millions of Muslim women across the world. However, despite the large body of literature that exists on the political and socio-cultural aspects of Islamic veiling, little is known about how the appearance of women wearing the hijab is perceived by other Muslim women within their native Muslim country. To throw light on this important issue, the current study focussed on the effects of the hijab on female facial attractiveness perceived by practising Muslim Emirati women living in their native Muslim country (the United Arab Emirates) who themselves wore the hijab as everyday attire. Participants were shown frontal-head images of women in three different conditions: covered (heads fully covered by the hijab except for the face), partially covered (heads fully covered by the hijab except for the face and the hair around the forehead) and uncovered (heads with no covering). The findings showed that faces in images where heads were covered and partially covered by the hijab were rated as equally attractive but both were rated as significantly less attractive than faces in images where heads were uncovered. These findings suggest that, even for practising Muslim Emirati females living in their native Muslim country for whom wearing the hijab is a normal aspect of everyday life, perception of facial attractiveness is compromised by wearing this garment. We argue that this effect of wearing the hijab is not consistent with a preference for one's own cultural group (cultural endogamy) and may, instead, occur because wearing a hijab occludes external features, such as hair and ears, which normally contribute to the perception of human facial attractiveness. In sum, while wearing the hijab may be dominated by male attitudes towards suppressing female attractiveness towards males, the findings from this study suggest that female Muslims too perceive the negative influence of wearing the hijab on female facial attractiveness.

## Introduction

The hijab (meaning *partition* or *barrier*) is worn as a traditional head-covering by millions of Muslim women throughout the world. For these women, the hijab is a visible expression of their faith and culture and a major determinant of being identified as Muslim. Indeed, wearing this item of traditional Muslim clothing appears to exert considerable influences on how others perceive the individuals concerned (e.g., [1]). Unfortunately, these perceptions are not always positive, and an often-discussed effect in non-Muslim “Western” societies is that perceptions of Muslim women wearing the hijab are frequently negative (for a review, see [2]) and wearing the hijab is likely to increase hostility and promote outgroup perceptions towards an individual in everyday life (for examples, see [3, 4, 5]).

**Fig 1:**
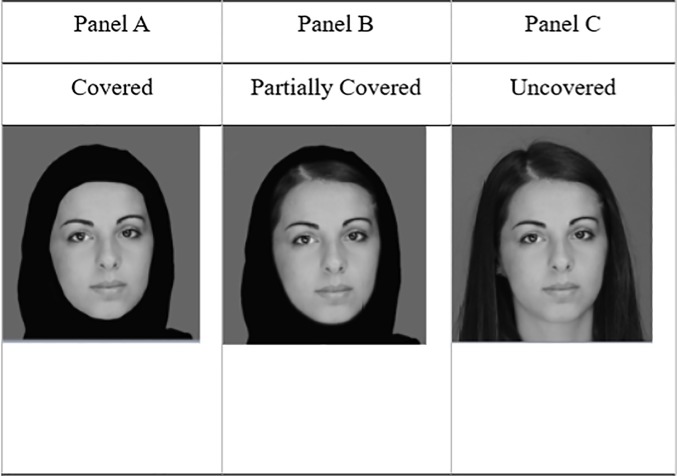
Examples of stimuli used in the three display conditions of this study.

However, the effect of wearing the hijab on the perception of Muslim women within a Muslim country is far from understood. Of particular importance is that the hijab is worn as a symbol of cultural identity, piety, and modesty, and Muslim women within a Muslim country, particularly in the United Arab Emirates (UAE), are encouraged to wear this head-covering when in public as a means of limiting their physical attractiveness to men [6]. Indeed, the way in which a woman wears a hijab (e.g., tightly around the face or more loosely, with some hair showing; see Fig 1, Panels A and B) is widely regarded as a public display of the depth of her faith and the extent to which she is intending to restrict her attractiveness. But despite these intentions, the effect of the hijab on how others perceive the facial attractiveness of the person wearing this item remains to be fully determined.

So far, several studies have investigated the effect of the hijab on various cognitive and perceptual processes, such as face recognition [7], memory for faces [8], and implicit bias [2]. But direct investigations of the effect of the hijab on perception of facial attractiveness are rare; we can find just three such studies in the literature and only one of these was conducted within a Muslim country. And yet facial attractiveness is associated with many aspects of human social perceptions, including priming the attribution of other positive characteristics such as honesty, intelligence, sincerity, moral virtue and overall competence (e.g., [9]). Furthermore, these perceptions have significant social consequences, to the extent that attractive women are seen as socially more appealing, occupy higher status jobs, marry higher status mates, and generally experience more upward economic mobility than their less attractive counterparts [9, 10]. Indeed the “what is beautiful is good” [10] stereotype has been found to extend even to the courtroom, with attractive women being less likely to be found guilty of a crime and receiving lighter punishments when they are actually convicted [9]. Clearly, therefore, a full understanding of the effect of wearing the hijab on facial attractiveness is extremely important for understanding the effect of the hijab on attitudes towards Muslim women.

Of the three studies investigating the effect of wearing the hijab on facial attractiveness that have been reported to date, two were conducted within a non-Muslim country (the UK [11, 12] hereafter M&S). Both these studies investigated perception of facial attractiveness by Muslim and non-Muslim British males who rated images in which women’s heads were displayed either entirely uncovered or covered by the hijab so that only each face was visible. Muslim and non-Muslim participants showed no significant difference in their attractiveness ratings for images where the hijab was worn, but non-Muslim males gave higher attractiveness ratings than Muslim males for images where women were uncovered. M&S suggest that these higher ratings were due to the location of the research (the UK) where negative perceptions of Islamic symbols, such as the hijab, place Muslims as outsiders and inspire negative attitudes towards individuals wearing this garment (e.g., [13]; see also [14]).

The hijab is an important marker of Muslim women's identity [15] and the results of M&S’s research with non-Muslim males provide an important indication of influences of cultural endogamy (a preference for one's own cultural group) on perception of others [16]. But although negative perceptions of Islamic symbols by male Westerners may explain why, in the research by M&S, non-Muslim males rated uncovered women as more attractive, the full influence of the hijab on facial attractiveness remains to be determined. Of particular relevance is that the ratings by Muslims in the studies by M&S were made by men. But the Hijab is often regarded as an example of male authority over female behavior which serves to maintain gender differences [17] and so it is uncertain how much the attractiveness ratings made by Muslim males were affected by these influences. In addition, as M&S point out, because the research was conducted in the UK, anti-Islamic feeling may have become internalized even by the Muslim males who took part, and this may have caused some moderation of their judgments of the attractiveness of hijab-wearing women in those earlier studies. Indeed, while not different significantly, images where the hijab was worn were rated as slightly less attractive by Muslim males than by non-Muslim males.

Some of these issues were addressed by a subsequent study by Pasha-Zaidi [6] in which Muslim females living in either a Muslim country (the UAE) or the USA were shown images of female faces wearing the hijab. Full-face photographs of Caucasian and South Asian women were used, one of each face wearing the hijab and one uncovered, and participants had to rate each image for facial attractiveness. The findings suggested that images of faces wearing the hijab were rated as more attractive by both sets of participants. But while these findings are interesting, several aspects of the study by Pasha-Zaidi suggest that the results they report may have been affected by other, confounding factors. The first of these is that the purpose of this study was to use only participants who originated from South Asia (mostly India, Pakistan, and Bangladesh). Whilst this would not necessarily be problematic for participants in the USA, participants in the UAE would have been non-native, ex-patriate residents with limited residents’ visas due to strict UAE laws concerning citizenship and residency. Moreover, social distinctions between ex-patriate occupants and native Emiratis are substantial in the UAE, and residents from South Asia generally take lower-ranking jobs, occupy lower levels on the social scale, and experience considerably less job security and greater risk of deportation if laws or customs are contravened [18]. Accordingly, as wearing the hijab is the norm in the UAE amongst native UAE Emirati females, the preference shown by UAE South Asian participants for images of female faces wearing the hijab in the Pasha-Zaidi study may reflect internalized self-preserving deference towards the prevailing Emirati culture. Indeed, the tendency to present oneself in a more favourable light can often lead to a social desirability bias [19] which undermines the validity of findings collected using self-reported measures such as surveys and interviews [20]. This possibility gains support from the finding of the Pasha-Zaidi study that whereas participants in the USA who did not themselves wear the hijab rated the attractiveness of hijab-wearing images lower than participants who wore the hijab, participants in the UAE rated hijab-wearing images higher with no evidence of a link to their own preference for wearing the garment.

These problems are compounded further by Pasha-Zaidi’s [6] use of online surveys to gather participants’ ratings of facial attractiveness as well as their personal details and background information. While this approach allowed large numbers of participants to take part with relatively little effort, it provided insufficient control over who actually completed the surveys and provided the attractiveness ratings (see also discussions by [21]). Indeed, this problem was exacerbated by including a snowballing technique in which additional participants for the study were selected simply by the participants already taking part. Finally, and of considerable concern, the facial images used in the experiment were not matched precisely across the two key stimulus conditions (hijab vs no hijab) as each face (10 in total) was photographed separately when wearing and not wearing the hijab. Under these circumstances, changes in facial expression and appearance can easily occur and this problem is visually apparent in the example images provided in the Pasha-Zaidi paper. Unfortunately, it is well known that even slight changes to the visual appearance of faces can alter their attractiveness substantially (e.g., [9, 22, 23, 24]) and so without precisely matching the facial images used in each condition, the actual effect of wearing a hijab on facial attractiveness cannot be determined.

Against this background, the purpose of the present study was to develop a greater knowledge of the effects of the hijab on perception of facial attractiveness by extending previous research in several key ways. First, in contrast to the work of M&S [11, 12], we investigated the effect of the hijab on perception of facial attractiveness by practicing Muslim women living in their native Muslim country (the UAE) where Islam and wearing the hijab are normal and widespread aspects of everyday life, and where anti-Islamic feelings should not influence participants’ judgments. Second, in contrast to the work of Pasha-Zaidi [6], all participants were native Emiratis whose personal details and background had been screened carefully for inclusion to ensure that their ratings would provide a genuine assessment of how the hijab is perceived by native Emiratis in the UAE. Finally, care was taken to match facial images precisely across covered and uncovered conditions to provide an accurate measure of facial attractiveness in each condition that was not contaminated by differences in facial expression (cf. [6]).

If cultural endogamy is a major determinant of facial attractiveness in hijab-wearing women, the influences of piety and devoutness to Islam within the UAE should lead our participants to rate the facial appearance of hijab-wearing women highly. In the work by M&S, attractiveness ratings made by British Muslims were positively correlated with religiosity for ratings of faces of females wearing a hijab but not for faces without. Moreover, the study of Pasha-Zaidi ([6] indicated an inverse relationship between religiosity and attractiveness of faces not wearing the hijab, such that UAE participants with higher levels of religiosity rated uncovered images as lower in attractiveness. Accordingly, to also examine if religiosity plays a role in perception of facial attractiveness in the present study, participants were also assessed for their religiosity using the Duke University Religion Index measure of religious involvement (DUREL; [25]). However, if perception of facial attractiveness by native Emirati women is not dominated by endogamy, and the status of our participants within their native country enables them to feel more confident in making assessments of facial attractiveness based on perceptual rather than cultural factors, the findings obtained should provide a more transparent indication of the effect of the hijab on facial attractiveness. Indeed, it may be the case that native, hijab-wearing Emirati women really do perceive the hijab as an effective limiter of female facial attractiveness and find uncovered facial images more attractive.

## Method

### Participants

Sixty females, aged 17–24 years, participated in the experiment and were recruited via flyers posted throughout Zayed University. All participants were native Emiratis born in the UAE, were practicing Muslims, and wore the hijab routinely in everyday life. These details were checked using official documentation and personal interviews to ensure that all participants fulfilled the requirements of the study. All participants also had normal or corrected-to-normal visual ability, as determined by Bailey-Lovie [26] assessments (see [27]).

### Ethics statement

This study was carried out in accordance with the recommendations of the Research Ethics Committee at Zayed University, with written informed consent from all participants, in accordance with the Declaration of Helsinki. The protocol was approved by the Research Ethics Committee at Zayed University. In addition, the individual shown in Fig 1 gave written informed consent to publish these images, as outlined in the relevant PLOS consent form.

### Stimuli

Photographs of the frontal views of 20 Muslim women of Middle Eastern appearance against a constant neutral background were used. Each woman routinely wore the hijab in everyday life and was photographed both uncovered and wearing her own hijab in the two styles used in the experiment. Three conditions were then constructed for each of the 20 heads (see Fig 1; N.B. The individual shown gave written informed consent to publish these images, as outlined in the relevant PLOS consent form). In the first condition (*covered*, Panel A), each head was fully covered except for the face. In the second condition (*partially covered*, Panel B), each head was also fully covered except for the face but now a small area of hair was also visible around the forehead. This is an alternative wearing of the hijab in the U.A.E. In the third condition (*uncovered*, Panel C), each head was completely uncovered. Each face was shown in each display condition. To avoid serious confounding differences in facial appearance that would otherwise occur across conditions, it was important to ensure that, for each woman, the same facial image was used for each of the 3 displays conditions. Accordingly, the uncovered image was used as the basis for each partially covered and covered image so that the hijab in each case was superimposed on the same facial image to produce 3 displays in which each face was shown unaltered except for the effect of the hijab manipulations. When asked at the end of the experiment, all participants reported that the 60 images looked natural and that they were unaware that the facial images used were sometimes identical.

### Apparatus and design

All 60 stimuli were presented in a different random order to each participant, shown full-size on a high-definition LCD monitor. Presentations and responses were controlled using Experiment Builder (SR Research Ltd., Kanata, Ontario, Canada) running on an Apple Macintosh 3.7 GHz.

### Procedure

Participants were tested individually and anonymously in a quiet room. At the start of their experimental session, each participant was informed that images of females would be displayed individually, each with a 7-point Likert scale which they should use to rate how attractive they regarded each face (1 = *not at all attractive* to 7 = *very attractive*). Each participant sat 60 cm from the screen and each image was presented until a response was made, after which the next image appeared. Participants began with five practice stimuli to familiarize themselves with the procedure before the experimental stimuli. After their experimental session, each participant rated their religiosity using the Duke University Religion Index measure of religious involvement [25] which measured intrinsic religiosity, degree of personal religious commitment, and degree of religious motivation of each of our participants. Participants’ responses were based on a 1 (*strongly disagree*) to 5 (*strongly agree*) response range.

## Results

**Fig 2:**
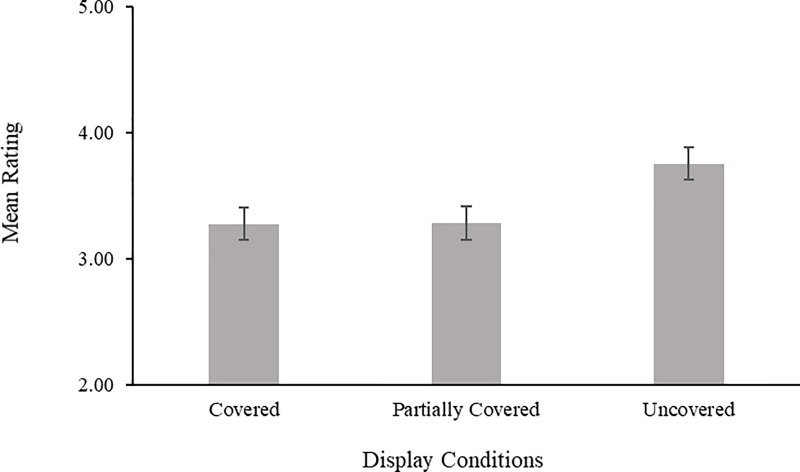
Mean attractiveness ratings for covered, partially covered, and uncovered images. Ratings in the experiment were on a scale of 1–7 and the scale here has been adjusted for clarity.

The mean attractiveness ratings for each display condition are shown in Fig 2. A repeated measures ANOVA of attractiveness ratings for covered, partially covered, and uncovered displays with a Greenhouse-Geisser correction showed a highly significant main effect, *F*(1.27, 75.04) = 23.99, *p* < .0001, η_p_^2^ = .29. Post-hoc comparisons using Bonferroni-corrected *t*-tests revealed that faces in images where heads were covered or partially covered were rated as equally attractive (3.28 vs 3.28, *p*>.90) but both were rated as significantly less attractive than faces in images where heads were uncovered (3.75, *p*s < .01).

On average, participants had a religiosity score of 4.40 out of a maximum score of 5 (SD = 0.49, range = 3–5, mode = 4.67), indicating that participants generally rated themselves as being highly religious. A Shapiro-Wilk test of distribution normality was significant, *S-W* = .90, *df* = 60, *p* < .0001, suggesting a skew towards high religiosity scores. Accordingly, a Spearman correlation coefficient was used to assess the association between the DUREL test scores and attractiveness ratings for the three display conditions and showed no significant correlations (for all displays, *r* < .12, *p*>.39). However, as religiosity scores generally were high, obtaining a correlation between attractiveness ratings and religiosity may have been adversely affected by celling effects. To examine this possibility, two groups of participants were formed, one containing 13 participants with the highest religiosity scores (all scores were either 4.67 or 5) and one containing 13 participants encompassing a wider range of religiosity scores (scores of 3, 4, & 5). Each group showed no significant correlation between attractiveness rating and religiosity for each of the three display conditions (for all displays in the highest group, *r* < .47, *p*>.10; for all displays in the wider-range group, *r* < .15, *p*>.60), suggesting that the overall absence of correlation between ratings and religiosity was not due to a ceiling effect. Moreover, when attractiveness ratings were compared across the two groups, an ANOVA with factors group and display condition showed only an overall main effect of display condition, *F*(2, 48) = 10.72, *p* < .0001, η_p_^2^ = .31 and no main effect of group, *F*(1, 24) = 0.27, *p*>.60, η_p_^2^ = .01 or interaction between the two factors, *F*(2, 48) = .55, *p*>.50, η_p_^2^ = .02. Thus, both groups produced similar patterns of perceived facial attractiveness despite differences in their religiosity scores and ranges.

## Discussion

The purpose of this study was to investigate perception of facial attractiveness in images of women either wearing the hijab or with their heads uncovered. Importantly, and for the first time in the published literature, the participants providing these perceptions were practicing Muslim women living in their native Muslim country (the UAE) where Islam and wearing the hijab are normal and widespread aspects of everyday life. Under these conditions, perceptions of facial attractiveness by Muslim women for women wearing the hijab were examined directly, and anti-Islamic feelings were unlikely to be present, thus circumventing negative influences on judgments of women wearing the hijab that may have been present in previous research conducted in the UK. But despite these expectations, and despite strong influences of religious conviction in Islam and the high levels of religiosity observed amongst our participants, practicing Muslim women in this indigenous Muslim culture showed a preference for the facial attractiveness of women not wearing the hijab.

Given the key characteristics of this study, it seems unlikely that the higher facial attractiveness ratings observed for uncovered images were due to negative perceptions of religious affiliation or influences of cultural endogamy. So why were these higher ratings observed? One initial possibility is that participants had an awareness of the intention of covered females to appear physically less attractive, and so the ratings of the images they were viewing reflected a form of self-fulfilling prophecy [28] or confirmation bias [29]. But in the UAE, fully-covered hijabis (women wearing the hijab) are widely regarded as showing a greater depth of faith than partially-covered hijabis and yet both types of image produced identical ratings of attractiveness in our study. Accordingly, if a cognitive bias of this type existed in our participants’ ratings, its influence appears to have been very weak indeed.

A more powerful explanation of why uncovered images produced higher ratings of facial attractiveness lies in the effect of the hijab on normal processes of facial perception. In particular, wearing a hijab obscures external features (such as hair and ears) which are naturally visible when viewing an unoccluded human face. Moreover, humans do not process faces as a collection of distinct facial features but rather as an integrated perceptual whole [30, 31], and external features, like hair and ears, play an important role in this process ([7, 32]; see also the external feature processing advantage [33]). For example, Toseeb and colleagues [7, 32] found that wearing the hijab produces substantial differences in the way uncovered faces are recognized, and concluded that the external features of a face play an important role in face recognition and that facial processing changes when these features are not visible.

Given this link between external facial features and facial processing, it seems likely that external features also play an important role in facial attractiveness and there is some evidence to provide support for this view [34]. In particular, Kramer and Ward presented female faces shown complete or with only their internal features present (e.g., eyes, nose, mouth) and found that external features contributed to the accurate discrimination of facial characteristics that are associated with facial attractiveness. Since wearing a hijab has substantial effects on the visibility of external features, therefore, it is plausible that, distinct from preferences that may be determined by culture, religion, and identity, wearing the hijab affects facial attractiveness by disrupting normal processes of facial perception. Indeed, the occlusion of hair may be a particularly influential component of the reduction in female attractiveness we observed. Many women spend a great deal of time and effort grooming their hair in order to appear more attractive (for discussions, see [35, 36]), and studies have shown that hair provides an observable indication of a woman’s health and youth and can add significantly to a woman’s attractiveness [37, 38]. Moreover, in the present study, both covered and partially covered images occluded similar amounts of hair, and this may explain why both types of hijab produced identical lower ratings of facial attractiveness. But again, both types of covered image produced identical ratings of attractiveness in our study despite the fact that, for native Emiratis in the UAE, fully-covered hijabis are regarded as showing a greater depth of faith than partially-covered hijabis. The effect of the hijab on occluding the external features of women’s faces, therefore, may be a profound and powerful perceptual influence on determining facial attractiveness.

The clear purpose of this study was to investigate perception of female facial attractiveness by native female Muslims but the perceptual interpretation of the effectiveness of the hijab on facial attractiveness may also provide new insight into the effect of religious veiling on regulating and restricting sexuality. As Pazhoohi, Lang, Xygalatas, and Grammer [39] point out (see also [40]), while the social and personal aspects of the cultural practice of veiling have been studied extensively, the actual function of this practice remains unknown. To help throw light on this issue, Pazhoohi et al. [39] sought to see, from an evolutionary perspective, whether an adaptive link exists between the use of religious veiling and ecological variation. More specifically, they argued that if the primary purpose of religious veiling is to guard female mates from rival males, an adaptive consequence of this purpose may be the greater use of veiling in harsh and demanding environments where the cost of paternal investment is higher, and so where more effort should be directed toward controlling and guarding female mates from infidelity. Their findings supported this argument. But while these decisions may be dominated by male requirements for female mate guarding, the findings from our study suggest that female Muslims can themselves perceive the influence of wearing the hijab on female attractiveness. Consequently, it should not be overlooked that Muslim females too may often wish to control their attractiveness towards men, and are aware that the hijab is effective for this purpose.

As a final point, it should be noted that the findings of the present study contrast strongly with those of Pasha-Zaidi [6], although both studies addressed the effect of the hijab on perception of facial attractiveness by participants living in the UAE. From the problems described earlier concerning the Pasha-Zaidi study, it is impossible to be certain about the underlying causes of this difference. On the one hand, it may be the case that native Emirati women and ex-patriate South Asian women living in the UAE really do have different perceptions about the effect of the hijab on facial attractiveness in the UAE, perhaps driven (as we suggested earlier) by internalized self-preserving deference by South Asians towards the prevailing Emirati culture. But in the absence of the necessary controls over participants, procedure, and facial stimuli in that earlier study, a more informative comparison between these two sets of findings is currently not possible.

In sum, the findings of the present study indicate that wearing the hijab reduces female facial attractiveness as perceived by other hijab-wearing Muslim women within their native Muslim country. Thus, while one intended purpose of the hijab is to limit women’s attractiveness to males, the findings we report indicate that this influence on attractiveness is also experienced by Muslim female observers, and so indicate that the hijab has a wider effect on the way in which covered Muslim women are perceived in society. However, given the nature of this study, it seems unlikely that this negative effect is the result of cultural endogamy on the perception of others. Instead, these effects of perceiving hijab-wearing women as facially less attractive may reflect normal processes of perceiving facial attractiveness and the negative influence on these processes when external features are concealed. Indeed, it remains to be seen if such influences contribute to the negative perceptions experienced by hijab-wearing women in some societies (for a review of these experiences, see [2]). In particular, it is widely accepted that a person rated high in attractiveness may also be perceived as high in social appeal, intelligence, employability, competence, and other major social factors. But the opposite may also be true, and negative influences on the normal processes of perceiving facial attractiveness produced when wearing the hijab may conceivably help inspire the hostility often experienced by hijab-wearing women in Western countries. Further research will cast yet more light on these important issues.

## Supporting information

S1 Table Data 60 Female Participants. (XLSX)
